# Biodegradation influence on alkylphenanthrenes in oils from Bongor Basin, SW Chad

**DOI:** 10.1038/s41598-019-49495-1

**Published:** 2019-09-10

**Authors:** Haiping Huang, Zhiqiang Li, Zhigang Wen, Denglin Han, Renfang Pan

**Affiliations:** 1 School of Geosciences, Yangtze University, Wuhan, 430100 Hubei P.R. China; 2 Department of Geoscience, University of Calgary, 2500 University Drive NW, Calgary, AB T2N 1N4 Canada; 3 The Key Laboratory of Exploration Technologies for Oil and Gas Resources (Yangtze University), Ministry of Education, Wuhan, Hubei 430100 China

**Keywords:** Biogeochemistry, Solid Earth sciences

## Abstract

Oil samples from the Bongor Basin, SW Chad have been geochemically characterized to investigate the biodegradation influence on alkylphenanthrenes. Concentrations of C0–3-alkylphenanthrenes (C0–3Ps) increase markedly after level 6 biodegradation due to the removal of other vulnerable components, decrease sharply after level 7 biodegradation and approach to absence at level 8. Phenanthrene appears to have higher ability to resist biodegradation than C1–3Ps at certain biodegradation levels (≤level 7) due to demethylation, which has been inferred as a possible reaction process during biodegradation of the aromatic hydrocarbons. The enrichment of non-alkylated phenanthrene in biodegraded oils makes biodegradation assessment complicated on the basis of alkylphenanthrene distributions. Individual isomers in alkylphenanthrenes exhibit variable ability to resist biodegradation influence. While certain isomers do show higher ability to resist biodegradation than others, no uniform biodegradation sequence can be established. Meanwhile, the biodegradation susceptibility between hopanes and alkylphenanthrenes varies greatly in different samples. The biodegradation systematics of alkylphenanthrenes proves to be highly complex, which may be indicative of the multiple charges and mixing during biodegradation.

## Introduction

Alkylphenanthrenes, characterized by fused-ring chemical structures, are the most common and abundant polycyclic aromatic hydrocarbons (PAHs) in source rock extracts and oils, which are originated from diagenesis and catagenesis alterations of sedimentary organic matter in depositional basins or combustion of fossil fuels and/or biomass. The abundance and distribution of alkylphenanthrenes are governed by both primary organic inputs and secondary alteration processes^1–3^.

Biodegradation transforms normal gravity light oil to heavy oil and bitumen by removal light hydrocarbons from oil in the reservoirs^4–9^. The effect of biodegradation can be assessed by using the occurrence and/or absence of certain hydrocarbon components, isomer distributions and relative abundance of compound classes. There are several artificial ranks available in literature^4,5,10–13^. The most commonly used one was proposed by Peters and Moldowan^4^ (abbreviated as ‘PM level’) with PM1 referring the lightest biodegradation and PM10 referring the heaviest biodegradation. However, various aromatic compound classes except triaromatic steroids have not been included in this biodegradation scales. Larter *et al*.^14^ noted that many samples from northern Alberta have been biodegraded to a uniform PM level but show great variable extent of alteration in aromatic compounds. They proposed the Manco (Modular Analysis and Numerical Classification of Oils) biodegradation scale by visual examination of compound completeness in various compound classes. It provides much higher resolution to differentiate biodegradation influence than commonly used scales and can recognize mixing of fresh oil with biodegraded oil and track oil property variation^14^.

The effects of biodegradation on alkylphenanthrenes distributions were well recorded by numerous studies^5,15–21^. It is widely accepted that the biodegradation rate decreases with increasing degree of alkylation, i.e., phenanthrene and methylphenanthrenes are removed before C2- and C3Ps are attacked^14,22,23^. While demethylation of alkylphenanthrenes to possibly form none alkylated phenanthrene has been proposed in our previous studies^15^, no case history further proves such concept. The methyl position in alkylphenanthrenes has different susceptibilities to resist biodegradation. The 9-methylphenanthrene (9-MP) is generally more refractory to biodegradation^15–17^, though some exceptional scenarios that 9-MP was removed prior to other isomers may exist^22^. Whether the biodegradation sequence of C2- and C3Ps established based on the observation from Liaohe oilfield^15^ exhibiting similar behavior as methylphenanthrene isomers in different basins has not been fully investigated in the literature.

There is no doubt that biodegradation exerts strong influence on the concentrations and distributions of alkylphenanthrenes, however, biodegradation of alkylphenanthrenes regarding PM levels seems ambiguous. Volkman *et al*.^11^ noticed that when 25-norhopanes present (at PM 6), no alkylphenanthrenes can be detected, while Huang *et al*.^15^ reported that alkylphenanthrenes can survive in a wide range of biodegradation scale up to PM 8. The biodegradation impact on different compound classes and their susceptibility actually require more systematic investigation. Alternatively, biodegradation signatures preserved in different compound classes from different basins may be controlled by different geological conditions, such as multiple changes and mixing, which may cause inconsistent and confusion biodegradation level assignment. Microbes likely consume various compounds synchronously at different rates^14^, rather than a sequential depletion^6,7^. Caution should be taken when compound distribution pattern was applied for biodegradation influence assessment.

Oil samples, suffered variable biodegradation influence from the Bongor Basin, SW Chad, have been geochemically characterized in the present study. The main objectives of our study are to elucidate the effects of biodegradation on alkylphenanthrenes concentrations and isomer distributions and to infer possible biodegradation mechanisms occurring in the reservoir.

## Sample Background and Experimental Methods

### Sample background

**Figure 1:**
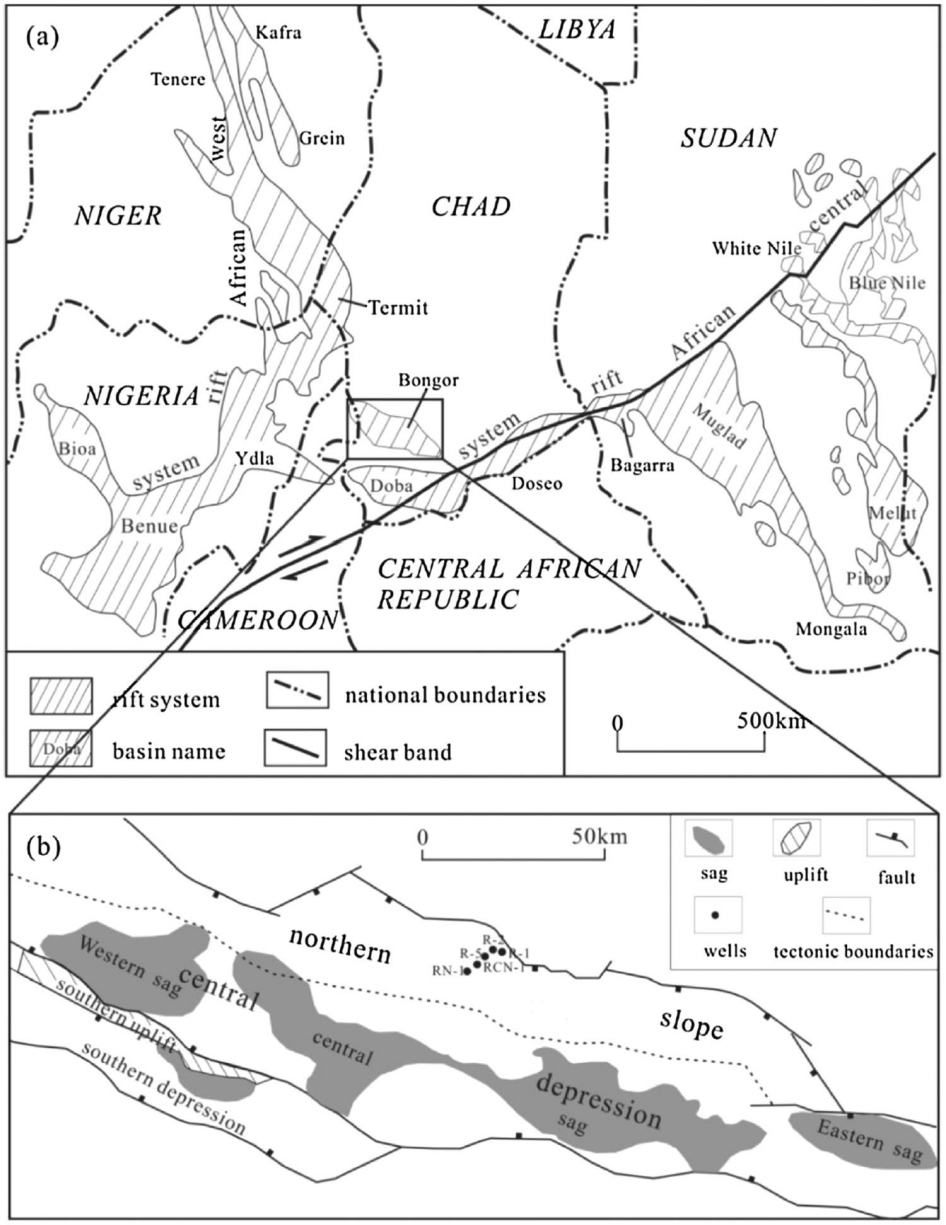
(**a**) West-central African Rift System and basin location. (**b**) Structural sketch map with sample locations in the Bongor Basin (modified from Song *et al*.^25^).

Bongor Basin is a prolific oil production province in the southwest of Chad. The basin length is about 280 km with an east-west extension and width is about 40–80 km with an area of 1.8 × 10^4^ km^2^. It is a rift basin formed during the Mesozoic–Cenozoic era by the influence of the Central Africa dextral strike-slip fault^24,25^ (Fig. 1a). The basin features with a half-graben structural style, faulted in the south and overlapped in the north. There are four secondary structural units in the basin: southern depression, southern uplift, central depression and northern slope (Fig. 1b). Currently, most oil accumulations have been discovered from the northern slope. The Ronier structure is a typical oil accumulation unit in the northern slope where all oil samples in the present study are situated.

The petroleum system elements of the Bongor Basin have been investigated by various early studies^25–27^. The Lower Cretaceous strata are developed in a continental fluvial-delta-lacustrine system with a maximum sedimentary thickness of >6 km which can serve as main source rock and reservoir assembly in the basin. The Upper Cretaceous has largely been eroded in most of the basin due to the orogeny activities by the end of the Cretaceous^28^. About 1.5 km thick of sediments has been eroded in the northern slope where oil reservoirs are elevated to a shallower depth and oils are suffered from variable biodegradation influence^27^. The oil source rocks are 500–1000 m thick dark mudstone formed under deep lacustrine facies during early Cretaceous, which are organic-rich with an average total organic carbon content of 3.5%, comprised mainly Type II kerogens and are highly matured. The non-biodegraded oils from deep reservoirs in the Bongor Basin are enriched in wax with an average wax content of 22.3%, poor in sulfur with an average sulfur content of 0.12% and poor in vanadium with an average V/Ni ratio of 0.1^25^, typifying a terrestrial origin. However, these biodegraded oils from the shallow reservoirs display variable bulk and molecular compositions, coupled with unusual high total acid number values (TAN, up to 8.3 mgKOH/g oil)^25,26^. Biomarker compositions of oils from the northern slope show very similar distribution patterns and can be correlated to a single source rock system developed at base of the Lower Cretaceous^25^. Therefore, changes in molecular compositions of oils are largely caused by biodegradation.

### Experimental methods

Fourteen (14) oil samples were collected from several exploration wells situated at Ronier structure (Fig. 1b). The asphaltene in a pre-weighed oil sample has been precipitated by adding coal hexane and filtered to obtain asphaltene content. The maltene fraction was then separated sequentially into saturated hydrocarbon fraction by using 50 ml of *n*-hexane as eluate and aromatic hydrocarbon fraction by using 50 ml of toluene as eluate on neutral alumina column chromatography. The internal standards of C_21_ sterane and d10-anthracene were added in saturated and aromatic hydrocarbon fractions, respectively, for quantitation purpose.

Molecular composition analysis of saturated and aromatic hydrocarbon fractions was performed on a HP 5973 mass spectrometer interfaced with a HP 6890 gas chromatography (GC) system. HP-5MS fused silica capillary column with 30 m length, 0.25 mm id and 025 μm film thickness has been selected. The GC oven temperature has been programmed from 60 °C to 150 °C with heating rate of 8 °C/min and then to 320 °C with heating rate of 4 °C/min. The selected ion monitoring (SIM) mode was operated in the mass spectrometer. The carrier gas in the GC column was helium and the flow rate was settled at 1 ml/min. The ion source of the mass spectrometer was operated in the electron ionization (EI) mode at 70 eV. Peak area was used for concentration calculation and no response factor calibration has been performed.

## Results

**Figure 2:**
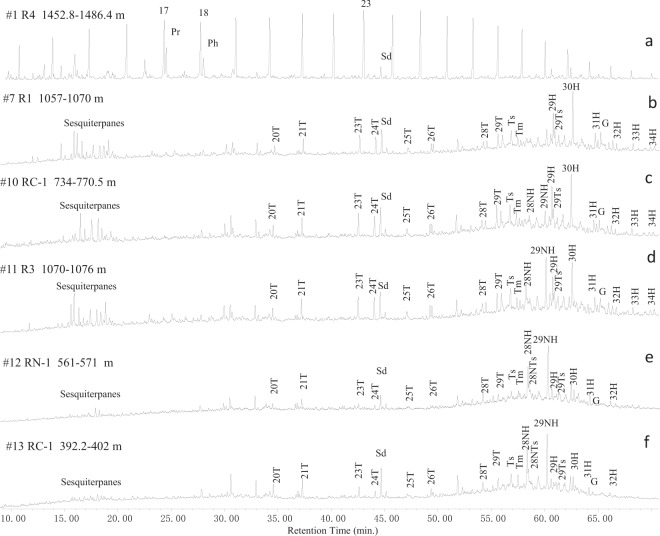
Representative total ion current chromatograms (TIC) of the saturated hydrocarbon fraction in the studied oils from the Bongor Basin. 17: carbon number of *n*-alkanes; Pr: pristane; Ph: phytane; 20–29 T: C_20–29_ tricyclic terpanes; Ts: 18α(H)-22,29,30-trisnorneohopane; Tm: 17α(H)-22,29,30-trisnorhopane; 28–29NH: C_28–29_ 17α(H), 21β(H) 25-norhopane; 29–34 H: C_29–34_ 17α(H), 21β(H) hopanes; 29Ts: 18-α(H)-30-norneohopane; G: gammacerane; sd: internal standard.

Representative reconstructed total ion chromatograms (TICs) of the saturated hydrocarbon fractions from studied oils show dramatic changes in the molecular compositions (Fig. 2). Six oils with burial depth greater than 1450 m (#1–6, Table 1) are characterized by the dominance of full suite of *n*-alkanes (Fig. 2a), showing no biodegradation influence, while 8 oils from reservoirs buried shallower than 1100 m (#7–14) are characterized by no *n*-alkanes or branched alkanes such as pristane and phytane, which had suffered from at least PM level 5 biodegradation influence (Fig. 2b–f). Among these biodegraded oils, samples #7–10 show relative enrichment of bicyclic sesquiterpanes (SST) in low molecular weight range and pentacyclic terpanes (PT) in high molecular weight range with C_30_ hopane as the highest peak on the TIC, suggesting PM level 6 biodegradation influence (Fig. 2b,c). While sample #11 still has intact bicyclic sesquiterpanes, the C_29_ 17α(H), 21β(H), 25-norhopane (C_29_NH) has higher apparent abundance than C_30_ 17α(H), 21β(H) hopane (C_30_H) on TIC, indicating more intensive biodegradation influence (PM level 7) (Fig. 2d). Samples #12–14 have suffered from the most severe biodegradation influence where sesquiterpanes and regular hopanes are largely removed with 25-norhopane as the dominant compounds on the TICs (PM level 8 or plus) (Fig. 2e,f). Tricyclic terpanes (TT) are mostly masked by *n*-alkanes in non-degraded oils, which are relatively concentrated and unaltered in samples #7–13 but have been degraded in sample #14 (PM level 8‒9).

**Table 1:** Molecular parameters derived from the saturated hydrocarbon fraction indicating maturity of and biodegradation influence on the studied oils from the Bongor Basin.

Sample #	Well	Depth (m)	TT/PT	TT/SST	Ts/(Ts + Tm)	20S/20(S + R)	29Ts/29 H	30D/30 H	29NH/30 H	28NH/29 H	PM Level
1	R4	1452.8–1486.4	0.56	0.52	0.41	0.42	0.24	0.09	0.00	0.00	0
2	R4-2	1578.2–1582.8	0.50	0.45	0.38	0.51	0.24	0.10	0.00	0.00	0
3	R4–6	1640–1643	0.43	0.96	0.43	0.40	0.25	0.07	0.00	0.00	0
4	RC-1	1613–1617	0.70	0.85	0.57	0.43	0.40	0.15	0.00	0.00	0
5	RCN-1	1610.5–1617.3	0.51	0.77	0.55	0.40	0.40	0.13	0.00	0.00	0
6	RCN-1	1894.9–1898	0.86	0.59	0.65	0.50	0.41	0.17	0.00	0.00	0
7	R1	1057–1070	0.60	1.06	0.63	0.46	0.49	0.19	0.10	0.18	6
8	R2	1066	0.53	0.95	0.62	0.41	0.44	0.17	0.04	0.06	6
9	R2	1078	0.62	0.92	0.62	0.43	0.51	0.20	0.16	0.31	6
10	RC-1	734–770.5	0.71	1.94	0.63	0.49	0.46	0.18	0.09	0.18	6‒7
11	R3	1070–1076	0.80	1.45	0.63	0.46	0.67	0.33	0.48	0.87	7
12	RN-1	561–571	1.40	2.90	0.59	0.46	1.42	0.95	1.69	2.13	7‒8
13	RC-1	392.2–402	1.62	3.80	0.62	0.44	1.62	0.68	3.03	7.36	8
14	RCN-1	1014.4–1024	2.21	3.80	0.49	0.56	1.44	0.72	4.73	5.37	8‒9

TT: tricyclic terpanes; PT: pentayclic terpanes; SST: sesquiterpanes; Ts: 18α(H)-22,29,30-trisnorneohopane; Tm: 17α(H)-22,29,30-trisnorhopane; 29Ts: 18-α(H)-30-norneohopane; 30D: C30 diahopane; 29–30 H: C_29–30_ 17α(H), 21β(H) hopanes; 28–29NH: C_28–29_ 17α(H), 21β(H) 25-norhopane.

The commonly used molecular parameters derived from the saturated hydrocarbon fraction are listed in Table 1. In non-degraded oils (#1–6), the summed tricyclic terpanes are less abundant than bicyclic sesquiterpanes and pentacyclic terpanes with TT/SST and TT/PT ratios less than 1.0. Due to the nature of highly resistant to biodegradation influence of tricyclic terpanes^29^, the ratios TT/SST and TT/PT increase sequentially in samples #7–14, suggesting increasing degrees of biodegradation influence. The commonly occurring 18α(H)−22,29,30-trisnorneohopane (Ts) and 17α(H)-22,29,30-trisnorhopane (Tm) in biodegraded oils appear to be more resistant to biodegradation than C_29–35_ regular hopanes. Ratios of Ts/(Ts + Tm) vary from 0.38–0.65 in non-degraded oils, while ratios are in the range of 0.49–0.63 in the biodegraded oils (Table 1). Narrow range of Ts/(Ts + Tm) ratios suggest similar maturity level of the studied oils. Similarly, C_29_ 20S/(20S + 20R) ratios fall in a narrow range of 0.40–0.51 for all oils except sample #14 where biodegradation has altered distribution of steranes (Table 1). The rearranged hopanes, especially 18-α(H)-30-norneohopane (C_29_Ts) and C_30_ diahopane (C_30_D) are present in minor amounts in non-degraded oils but relative concentrated in biodegraded oils. The ratios of C_29_Ts to C_29_ 17α(H), 21β(H) hopane (C_29_H) are <0.41 in non-degraded oils and increase to 1.62 in severely biodegraded sample. Similarly, the ratios of C_30_D to C_30_H are <0.17 in non-degraded samples and increase to 0.95 in heavily degraded sample (Table 1). The 25-norhopanes are commonly used to indicate intensive biodegradation influence, though they are not found in all heavily biodegraded oils. Ratios C_29_NH/C_30_H and C_28_NH/C_29_H increase from 0 up to 4.73 and 5.37, respectively, in heavily biodegraded oils (Table 1). Based on the occurrence of 25-norhopanes and partial/full removal of regular steranes and hopanes, biodegradation levels can be assigned accordingly in spite of the ambiguity remaining in some samples due to the simultaneous removal of regular steranes and hopanes (Table 1).

**Figure 3:**
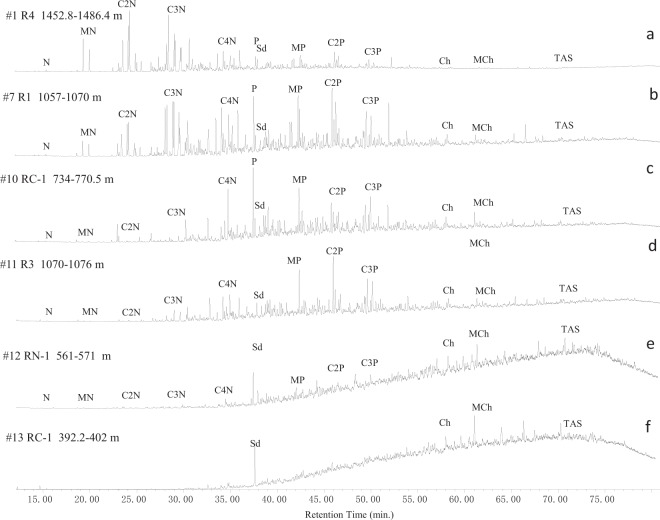
Representative total ion current chromatograms (TIC) of the aromatic hydrocarbon fraction in the studied oils from the Bongor Basin. N: naphthalene; MN: methylnaphthalenes: C2N: C2-alkylnaphthalenes; C3N: C3-alkylnaphthalenes; C4N: C4-alkylnaphthalenes; P: phenanthrene; MP: methylphenanthrenes; C2P: C2-alkylphenanthrenes; C3P: C3-alkylphenanthrenes; Ch: chrysene; MCh: methylchrysenes; TAS: triaromatic steroid hydrocarbons; sd: internal standard.

The TICs of the aromatic hydrocarbon fraction also exhibit a dramatic change in the studied oils (Fig. 3). The alkylnaphthalenes are the dominated aromatic compounds in non-degraded oils (Fig. 3a), while alkylphenanthrenes are relative enriched in the moderately biodegraded oils (samples # 7–9) (Fig. 3b). Sample #10 has overall similar TIC with samples #7–9 in the saturated hydrocarbon fraction but very different TIC in the aromatic hydrocarbon fraction where alkylnaphthalenes are largely depleted and alkylphenanthrenes are heavily altered (Fig. 3c). Sample #11 has suffered more intensive biodegradation than sample #10 evidenced by the removal of phenanthrene and severely altered alkylnaphthalenes (Fig. 3d). Both alkylnaphthalenes and alkylphenanthrenes are depleted in samples #12–14 where the chrysene and its methylated homologue become the dominated components in the TICs (Fig. 3e,f).

The relative abundance of identified aromatic compound classes in the studied oils are listed in Table 2. Alkylnaphthalenes are the most abundant homologs in non-degraded oils, which constitute 55.5–66.7% of the total quantified aromatic compounds, while the proportions of alkylphenanthrenes vary in the range from 20.8 to 32.4%. Other compound classes including biphenyl, dibenzothiophene, pyrene, chrysene and triaromatic steroid and their alkylated homologues are generally low with relative proportions of below 5% individually (Table 2). The relative abundance of alkylnaphthalenes declines to the range of 36.5 to 43.5% while the relative abundance of alkylphenanthrenes increases to 39.9 to 42.9% in samples #7–9. Biodegradation seems relatively concentrated the proportion of alkylphenanthrenes, tetracyclic aromatic hydrocarbons and triaromatic steroids by virtue of the loss of alkylnaphthalenes. The relatively proportion of alkylnaphthalenes further decreases in samples #10–11, while the proportion of alkylphenanthrenes increases continuously. Significant depletion of alkylphenanthrenes occurs in samples #12–13 where alkylnaphthalenes are almost completely destroyed. All 1‒3 ring aromatic components can hardly be detected in sample #14, where chrysene and methychrysenes become the dominant compounds (Fig. 3, Table 2).

**Table 2:** Relative abundance of identified aromatic hydrocarbon compound classes in the studied oils from the Bongor Basin.

Sample #	C0–5N	C0–3P	C0–1BP	C0–2DBT	C0–1DBF	C0–1F	C0–1Ch	C0–1Py	TAS
	(%)	(%)	(%)	(%)	(%)	(%)	(%)	(%)	(%)
1	63.3	20.8	3.9	2.3	2.7	4.6	1.2	0.4	0.3
2	66.7	23.5	1.5	1.2	1.1	2.4	2.2	0.4	0.5
3	58.6	30.5	0.8	1.3	0.9	2.4	3.1	0.3	1.3
4	55.5	32.1	1.3	1.6	1.2	3.2	2.7	0.4	1.6
5	63.3	25.3	1.3	1.2	0.9	2.4	2.0	0.3	2.8
6	56.8	32.4	1.9	1.5	1.0	3.6	1.7	0.8	0.3
7	38.4	42.9	0.9	1.8	1.2	6.0	4.8	2.1	0.9
8	43.5	39.9	1.1	1.6	1.3	5.4	4.1	1.8	0.7
9	36.5	42.0	1.0	2.0	1.3	7.2	5.2	2.6	1.2
10	11.2	52.8	0.5	3.4	2.3	14.4	9.2	4.1	0.9
11	18.5	48.3	0.3	3.3	1.8	10.9	8.4	4.1	2.6
12	6.9	23.5	0.6	3.7	1.5	6.1	23.7	10.5	16.7
13	8.0	23.9	0.4	1.8	1.2	6.0	33.0	4.2	14.7
14	1.2	4.6	0.3	0.0	0.2	0.1	72.5	2.8	9.4

C0-5N: C0–5-alkylnaphthalenes; C0-3P: C0-3-alkylphenanthrenes; C0-1BP: biphenyl and methylbiphenyls; C0-2DBT: C0-2-alkyldibenzothiophenes; C0-1DBF: dibenzofuran and methyldibenzofurans; C0-1F: fluorene and methylfluorenes; C0-1Ch: chrysene and methylchrysenes; TAS: triaromatic steroid hydrocarbons.

**Figure 4:**
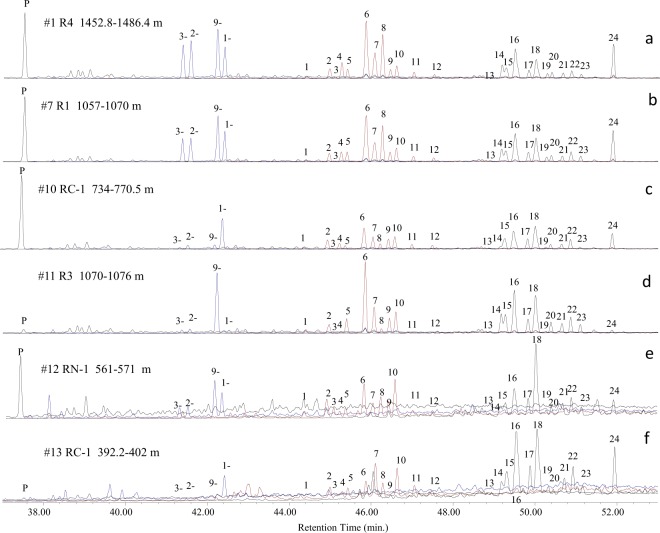
Representative summed mass chromatograms of alkylphenanthrenes (m/z 178, 192, 206, 220) in the studied oils from the Bongor Basin. P: phenanthrene; 3-: 3-methylphenanthrene (MP); 1: 3-ethylphenanthrene (EP); 2: 2-EP + 3,6- dimethylphenanthrene (DMP); 3: 9-EP + DMP; 4: 1-EP + 2,6 + 3,5-DMP; 5: 2,7-DMP; 6: 2,10 + 1,3 + 3,10 + 3,9-DMP; 7: 1,6 + 2,9 + 2,5-DMP; 8: 1,7-DMP; 9: 2,3-DMP; 10: 4,9 + 4,10 + 1,9-DMP; 11: 1,8-DMP; 12: 1,2-DMP; 13: trimethylphenanthrene (TMP); 14 + 15: 1,3,6- + 1,3,10- + 2,6,10-TMP + 2-EP-5-MP; 16: 1,3,7- + 2,6,9- + 2,7,9-TMP + 7-EP-1-MP; 17: 1,3,9- + 2,3,6-TMP; 18: 1,6,9- + 1,7,9- + 2,3,7-TMP; 19: 1,3,8-TMP; 20: 2,3,10-TMP; 21: TMP; 22: 1,6,7-TMP; 23: 1,2,6-TMP; 24: 1,2,8-TMP.

Figure 4 shows the representative mass chromatograms of the C0‒3 Ps (m/z 178 + 192 + 206 + 220) in the studied oils. The apparently intact distribution in samples #7–9 shows similar C0‒3P distribution pattern as these in non-degraded oils (samples #1–6) (Fig. 4a,b), while biodegradation exerts significant impacts on the distributions of all alkylphenanthrene homologues in heavily biodegraded oils (samples #10–14) (Fig. 4c–f). Interestingly, sample #10 shows slightly altered C3Ps, moderately altered C2Ps, heavily altered C1Ps but intact phenanthrene. Among 4 methylphenanthrene isomers, 3-, 2- and 9-methylphenanthrene (MP) were large destroyed, but 1-MP remain intact. In C2Ps, the loss of 1,7-dimethylphenanthrene (DMP) (peak 8) is more obvious than other isomers (Fig. 4c). Sample #11 seems show a ‘normal’ biodegradation consequence where phenanthrene has been removed before methylphenanthrenes and 9-MP shows the highest ability to resist biodegradation influence. The apparent loss of 1,2,8-trimethylphenanthrene (TMP, peak 24) occurs in the C3Ps (Fig. 4d). While samples #12–14 are suffered extreme level of biodegradation influence, their alkylphenanthrenes still display very different distribution patterns. Sample #12 has severely altered C1‒3Ps but relatively intact phenanthrene (Fig. 4e), however, phenanthrene can hardly be detected in samples #13. The 1,2,8-TMP has been largely depleted in samples #10–12 but remains high in sample #13 (Fig. 4f). Almost all alkylphenanthrenes have been removed in sample #14 (chromatograms not shown).

Concentrations of alkylphenanthrene homologues are listed in Table 3. All non-degraded oils have quite similar concentrations of alkylphenanthrenes with an average phenanthrene, C1P, C2P and C3P concentrations of 92, 259, 452 and 484 μg/g oil, respectively in sample #1–6 (Table 3). Samples #7–9 are suffered PM level 6 biodegradation influence but their concentrations of phenanthrene, C1P, C2P and C3P are much higher than these in non-degraded oils with an average value of 343, 601, 922 and 911 μg/g oil, respectively. Sample #10 has the highest phenanthrene concentration of 407 μg/g oil in all studied samples, while its C1P, C2P and C3P concentrations are much lower than these in samples #7–9. Sample #11 has slightly higher C1P, C2P and C3P concentrations than these in sample #10 but its concentration of phenanthrene is only 16 μg/g oil. Sharp decrease of concentrations in all alkylphenanthrene homologues occurs in sample #12, whereas concentrations of alkylphenanthrene components approach to noise level in samples #13–14 (Table 3).

**Table 3:** Concentrations, relative abundance of homologue ratios of alkylphenanthrenes in the studied oils from the Bongor Basin.

Sample #	P	MP	C2P	C3P	P	MP	C2P	C3P	MP/P	C2P/P	C3P/P	C2P/MP	C3P/MP	C3P/C2P
	(μg/g oil)	(μg/g oil)	(μg/g oil)	(μg/g oil)	(%)	(%)	(%)	(%)						
1	119	290	410	337	10.3	25.0	35.5	29.2	2.4	3.4	2.8	1.4	1.2	0.8
2	97	242	405	414	8.4	20.9	35.0	35.7	2.5	4.2	4.3	1.7	1.7	1.0
3	92	284	642	937	4.7	14.5	32.8	47.9	3.1	7.0	10.2	2.3	3.3	1.5
4	85	274	461	424	6.8	22.0	37.1	34.1	3.2	5.5	5.0	1.7	1.5	0.9
5	67	195	353	404	6.6	19.1	34.6	39.7	2.9	5.2	6.0	1.8	2.1	1.1
6	92	269	440	388	7.7	22.6	37.0	32.6	2.9	4.8	4.2	1.6	1.4	0.9
7	368	657	1019	1007	12.1	21.5	33.4	33.0	1.8	2.8	2.7	1.6	1.5	1.0
8	401	676	993	940	13.3	22.5	33.0	31.2	1.7	2.5	2.3	1.5	1.4	0.9
9	261	470	754	787	11.5	20.7	33.2	34.7	1.8	2.9	3.0	1.6	1.7	1.0
10	407	225	528	730	21.5	11.9	28.0	38.6	0.6	1.3	1.8	2.4	3.3	1.4
11	16	274	669	842	0.9	15.2	37.2	46.8	17.5	42.7	53.7	2.4	3.1	1.3
12	20	27	68	97	9.5	12.7	32.1	45.7	1.3	3.4	4.8	2.5	3.6	1.4
13	1	5	25	57	1.0	5.3	28.6	65.1	5.0	27.3	62.2	5.4	12.3	2.3
14	2	3	5	10	7.7	14.8	25.0	52.4	1.9	3.2	6.8	1.7	3.5	2.1

P: phenanthrene; MP: methylphenanthrenes; C2P: C2-alkylphenanthrenes; C3P: C3-alkylphenanthrenes.

## Discussion

### Degree of alkylation

The degree of alkylation is one of critical factors controlling the biodegradation rate of alkylphenanthrenes. It is widely accepted that the higher number of alkyl substituents the alkylphenanthrenes have, the less susceptible to biodegradation^11,15,18,23^. However, our previous studies revealed the exceptions to such rule when the relative abundances of the C2- and C3-alkylnaphthalenes (C3Ns) were compared. The ratio of C2Ns/C3Ns increased with increasing levels of biodegradation, suggesting possible demethylation of the C3Ns to form C2Ns during the advanced stages of biodegradation^15^. Nevertheless, demethylation as one of the mechanisms for aromatic hydrocarbon biodegradation has not been demonstrated and recognized in other case histories.

The demethylation of highly alkylated phenanthrenes to form low degree of alkylation counterparts can be illustrated by both concentrations and relative abundance of alkylphenanthrene homologues in oils from the Bongor Basin. Average concentration of phenanthrene increased from 92 μg/g oil in non-degraded oils (#1–6) to 343 μg/g oil in oils at PM 6 level of biodegradation (#7–9) with 3.7 times of increment, whereas the concentrations of C1‒3Ps increased 2.3, 2.0 and 1.9 times, respectively (Table 3). While all these alkylphenanthrene homologues were relatively concentrated due to the removal of other vulnerable compounds, the highest increment of phenanthrene concentration suggested additional source of input. As biodegradation was the main cause resulting in the change of component concentrations in oil, demethylation of C1‒3Ps to form non-alkylated phenanthrene was likely in process. The relative proportion of phenanthrene in whole C0‒3Ps increased from 7.4% in non-degraded oils (#1–6) to 12.3% in oils at PM 6 level of biodegradation (#7–9) (Table 3). Ratios of C1‒3Ps to phenanthrene decreased from 2.8, 5.0 and 5.4 in non-degraded oils (#1–6) to 1.8, 2.7 and 2.7 in oils at PM 6 level of biodegradation (#7–9), respectively, suggesting all these alkylphenanthrenes are biodegraded faster than phenanthrene. Intestinally, the ratios of C2- and C3Ps to C1Ps also decreased slightly from 1.7 and 1.9 in non-degraded oils (#1–6) to 1.5 and 1.5 in oils at PM 6 level of biodegradation (#7–9), respectively, suggesting that formation of methylphenanthrenes by cleavage of a methyl group from C2- and C3Ps is also likely in process. Demethylation of alkylphenanthrenes to form non-alkylated phenanthrene reached the extreme level in sample #10 where methylphenanthrenes were severely altered but phenanthrene had the highest concentration of 407 μg/g oil and highest relative proportion of 21.7% in all studied samples. However, demethylation of alkylphenanthrenes seem not obvious in sample #11 where phenanthrene was largely depleted together 3-, 2- and 1-MPs but C2- and C3Ps were relatively less affected. The concentration of phenanthrene was only 16 μg/g oil and its relative proportion was 0.9%. Biodegradation of this particular sample seem fit the alkylation degree impact rule. However, while sample #12 has suffered more intensive biodegradation influence than sample #11 as indicated by the dominance of 25-norhopanes in the saturated hydrocarbon fraction and more depleted C2- and C3Ps, the relative proportion of phenanthrene in sample #12 was still higher than these in non-degraded oils. The demethylation of alkylphenanthrenes to form phenanthrene can be illustrated by unusually low relative proportion of C1Ps in this sample. Microbial demethylation was obviously complicated by the possibility of the simultaneous biodegradation of parent compound and alkylated compounds at PM level 7. Nevertheless, when biodegradation approaches to more advanced stage (PM level > 7), the C3Ps are more recalcitrant to biodegradation than less alkylated counterparts.

Our study suggests that demethylation is an important step in alkylphenanthrene biodegradation especially for the formation of non-alkylated phenanthrene while the controlling factors for such reaction are still not clear. Demethylation of highly alkylated aromatic hydrocarbons to form low degree of alkylation counterparts may cause misjudgment of biodegradation influence especially when the chromatograms are simply used. In a Monco scale^14^, five increasing levels of biodegradation (0–4) have been described as ‘pristine’, ‘light’, ‘moderate’, ‘heavy’ and ‘depleted’ for each compound class. The light biodegradation (Manco level 1) of alkylphenanthrenes was characterized by altered phenanthrene and 1-MP but unaltered C2Ps, the Manco level 2 was characterized by altered C0‒2Ps with some resistant isomers, the Manco level 3 was characterized by removal of most of the C2P isomers and Manco level 4 contained no residual C0‒3Ps. Sequential biodegradation from low degree of alkylation to high degree of alkylation has been formatted in Manco scale calculation. However, if demethylation plays the role, the Manco score assignment would be complicated and might cause some confusion. Concentration coupled with visualization diagnosis might be better solution for biodegradation influence assessment.

### Position of substitute

**Figure 5:**
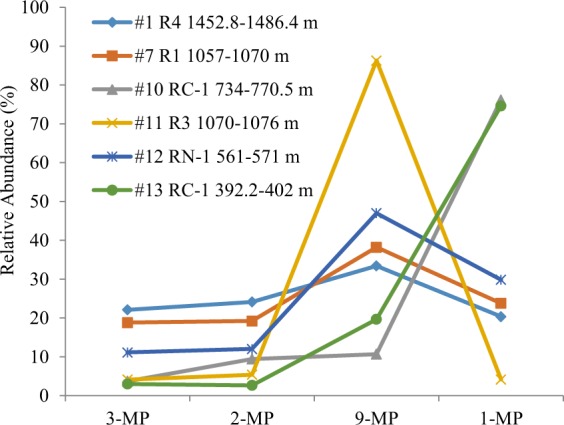
Relative abundance of methylphenanthrene isomers in oils from the Bongor Basin.

Studies of naturally biodegraded oils and laboratory experiments indicated that of the methylphenanthrenes, 9-MP is more resistant to biodegradation than other MP isomers^16,17,30^. Case histories from the Liaohe Basin indicated that relative abundance of 9-MP shows a consistent increase with increasing degree of biodegradation, with the most pronounced changes occurring at PM levels 5‒7. However, Bennett and Larter^22^ reported that 9-MP has been removed prior to 1-MP and both 9-MP and 1-MP might be occasionally removed before 3- and 2-MPs. Oils from the Bongor Basin may provide the further observation of methylphenanthrene isomer selectivity during biodegradation. Samples #1–9 have intact alkylphenanthrene distributions and all methylphenanthrene isomers have very similar relative proportions. Samples #10–13 have very different isomer distributions (Fig. 5). Sample #10 and #13 have 1-MP dominated in m/z 192 chromatograms where 1-MP accounts about 75% of total methylphenanthrenes, whereas sample #11 shows the predominance of 9-MP which accounts for 86% of total methylphenanthrenes. Furthermore, sample #12 has elevated 9- and 1-MPs but depleted 3- and 2-MPs (Fig. 5). The present study suggests that either 9-MP or 1-MP or both 9- and 1-MPs may show selective resistance to degradation compared to other methylphenanthrene isomers. While the ratio of (9- + 1-MP)/(3- + 2-MP) may show good response to increasing levels of biodegradation, no fixed order of susceptibility to biodegradation in methylphenanthrene isomers can be established.

**Figure 6:**
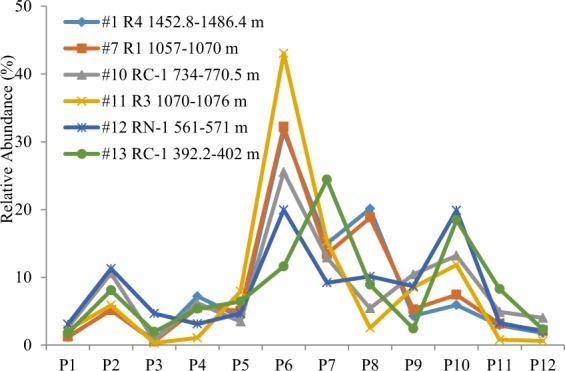
Relative abundance of C2-alkylphenanthrene isomers in oils from the Bongor Basin (see Fig. 4 for peak identification).

Our previous naturally biodegraded case history study illustrated that 1,7-DMP was the most vulnerable compound while 1,3- + 3,9- + 2,10- + 3,10-DMP was the most recalcitrant component^15^. The present study basically shown a similar C2P biodegradation order as that in the Liaohe oils but with some exceptions (Fig. 6). The relative proportion of 1,7-DMP in total C2Ps decreased from >20% in non-degraded oils to 2.5%, while relative proportion of 1,3- + 3,9- + 2,10- + 3,10-DMP in total C2Ps increased from ~30% in non-degraded oils up to 43% in sample #11. However, the decrease relative proportion of 1,7-DMP and increase of 1,3- + 3,9- + 2,10- + 3,10-DMP displayed a weak correlation with levels of biodegradation. Samples #10, 12 and 13 had much lower 1,3- + 3,9- + 2,10- + 3,10-DMP relative proportion than non-degraded oils, while their 1,7-DMP remained in decent proportion (Fig. 6). The susceptibility order to biodegradation seems variable in different samples.

**Figure 7:**
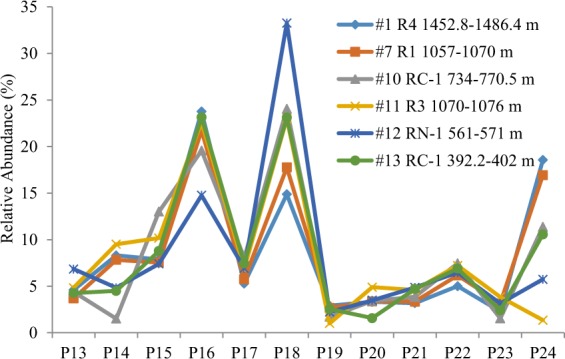
Relative abundance of C3-alkylphenanthrene isomers in oils from the Bongor Basin (see Fig. 4 for peak identification).

Similarly, a tentative order of susceptibility to biodegradation for the C3Ps had been established in the Liaohe case history with 1,2,8-TMP as the most susceptible one and 1,3,7- + 2,6,9- + 2,7,9-TMP + 7-E-l-MP as the least susceptible one^15^. However, biodegradation behavior in oils from the Bongor Basin shows some inconsistence with this order. The 1,2,8-TMP (peak 24 in Fig. 4) had the highest relative abundance of 26.6% (as percentage of the sum of C3Ps) in non-degraded oils (#1–6) and its proportion decreased to 17.3% at PM 6 level of biodegradation in samples #7–9. The lowest relative abundance of 1,2,8-TMP (1.4%) occurred in sample #11, which was suffered PM level 7 of biodegradation influence, whereas the relative abundance of 1,2,8-TMP increased in samples #12 and 13 as biodegradation approaches to more intensive stages. Similarly, 1,3,7- + 2,6,9- + 2,7,9-TMP + 7-E-l-MP (peak 16 in Fig. 4) was relatively concentrated in samples #11 and 13 but depleted in samples #12 (Fig. 7). The 1,6,9- + 1,7,9- + 2,3,7-TMP (peak 18 in Fig. 4) seemed have the highest ability to resist biodegradation in oils from the Bongor Basin. However, we caution against to establish the susceptibility order as the complex charge history may be involved in the oil fields. An order of biodegradation susceptibility in compound classes is a somewhat illusory concept as it depends not only on stereochemistry and thermodynamic considerations but also on, for example, overall oil composition, nutrient availability, redox conditions and the species composition in the microbial consortium. The situation obviously becomes much more complex in reservoirs containing a mixture of biodegraded and non-degraded oils.

### Hopane vs alkylphenanthrenes

Biodegradation schemes applied to indicate the level of degradation that a petroleum accumulation may have experienced are based on the sequential removal of hydrocarbons. While consequence of biodegradation influence on alkylphenanthrenes has been investigated for decades, the initiation and completion of alkylphenanthrene biodegradation as compared to the most commonly referenced hopanes remains uncertain^10–15^. Volkman *et al*.^11^ noted almost all of the alkylphenanthrenes have been removed by biodegradation when 25-norhopanes occur in oils at PM level 6. Larter *et al*.^14^ also suggested that great variable extent of alteration in aromatic compounds occur at one uniform PM level. However, Huang *et al*.^15^ observed much early biodegradation at PM level < 4 and wide range up to PM 8 in alkylphenanthrene biodegradation. This is probable reason why the most widely used PM biodegradation scales^4^ has not included in aromatic hydrocarbons except triaromatic steroids. The case from the Bongor Basin likely creates more complicated situations but helps to further elucidate biodegradation mechanisms and controlling factors.

In contrast to previous studies that concentrations of alkylphenanthrenes decreased before isomer distribution alteration^15^, concentrations of alkylphenanthrenes increased substantially in the oils from the Bongor Basin at PM level 6 when 25-norhopanes occur even though the distribution of alkylphenanthrenes was partially altered. Significant biodegradation of alkylphenanthrenes started at PM level 7 where hopanes were largely removed and 25-norhopanes became the dominant compound class in saturated hydrocarbon fraction. Alkylphenanthrenes were completely removed in sample #14 when tricyclic terpanes were degraded at PM level 8–9. Biodegradation of alkylphenanthrenes in oils from the Bongor Basin occurred later than these observed from the Liaohe Basin in terms of hopane biodegradation^15^, covered wider range of PM levels than these observed from the Western Canada Sedimentary Basin^14^ and shown different isomer selection orders from other case histories and laboratory simulations^15,17,19,23^. Similarly, Bennett and Larter^22^ noted that degradation of steranes, diasteranes and hopanes occurred simultaneously along with the formation of 25-norhopanes in the Athabasca oil sands, which might cause the confusion for PM level assignment especially at PM levels 6‒9. Therefore, to compare susceptibility of biodegradation between hopanes and alkylphenanthrenes or put changes of alkylphenanthrenes on PM biodegradation level is very complicated or even impossible. However, while PM level assignment remains practical in biodegradation assessment, inconsistency of biodegradation preferential between hopane and alkylphenanthrenes is probably norm due to multiple charges and mixing commonly occurring in biodegraded fields. The varied alkylphenanthrene biodegradation profiles in literature and oils from the Bongor Basin may be indicative of the relative impact of local factors arising from charge history, water chemistry, bitumen composition, mineralogy, microbiology and anaerobic biodegradation pathways, which are complicated by multiple changes and mixing processes^7,9,13,22^.

## Conclusion

Biodegradation exerts the primary control on alkylphenanthrene concentrations and distributions in oils from the Bongor Basin, SW Chad. Concentrations of alkylphenanthrenes increased significantly from non-degraded oils to oils biodegraded at PM level 6 due to the removal of other vulnerable components while sharp decrease of alkylphenanthrene concentrations ocurred at PM level 7. Phenanthrene had higher increment of concentration and relative abundance than C1‒3Ps at PM level 6 and decreased much slower at PM level 7. Demethylation of a substituted compound was inferred as an important step in the aromatic hydrocarbon biodegradation process while rule of degree of alkylation impact might still work at PM level 8 before complete removal of alkylphenanthrenes. The enrichment of the non-alkylated phenanthrene caused by cleavage of methyl groups from highly alkylated components challenges biodegradation impact assessment and results in confused biodegradation level assignment. Concentration rather than chromatogram alone may help to decipher the complicated situations. The biodegradation of the C1–3P isomers shown great variability and no uniform susceptibility order can be established. Using hopane series as reference, biodegradation behaviors of alkylphenanthrenes in oils from the Bongor Basin differ significantly from these reported in the literatures. The inconsistency of biodegradation preferential order in alkylphenanthrene isomers and variable resistant ability as compared to hopanes observed in the present study emphasize that local factors arising from charge history, water chemistry, microbial consortium and anaerobic biodegradation pathways exert the dominant controls on the susceptibility of alkylphenanthrenes during biodegradation. No uniform biodegradation behavior should be expected under different basin regimes and conditions.
